# Magnetic resonance imaging in acute meningoencephalitis of viral and unknown origin: frequent findings and prognostic potential

**DOI:** 10.3389/fneur.2024.1359437

**Published:** 2024-01-17

**Authors:** Lena S. Abbuehl, Mattia Branca, Anamaria Ungureanu, Andrea Federspiel, Stephen L. Leib, Claudio L. A. Bassetti, Arsany Hakim, Anelia Dietmann

**Affiliations:** ^1^Department of Neurology, Inselspital, Bern University Hospital, University of Bern, Bern, Switzerland; ^2^CTU Bern, University of Bern, Bern, Switzerland; ^3^Support Center for Advanced Neuroimaging Translational Imaging Center (sitem-insel), Institute for Diagnostic and Interventional Neuroradiology, Bern University Hospital, University of Bern, Bern, Switzerland; ^4^Institute for Infectious Diseases, University of Bern, Bern, Switzerland; ^5^Institute of Diagnostic and Interventional Neuroradiology, Inselspital, Bern University Hospital, University of Bern, Bern, Switzerland

**Keywords:** viral meningoencephalitis, encephalitis, prognosis, MRI, tick-borne encephalitis

## Abstract

**Background:**

Magnetic resonance imaging (MRI) findings in meningoencephalitis have mainly been described in terms of their diagnostic value rather than their prognostic potential, except for herpes simplex virus (HSV) encephalitis. The aims of our study were to describe frequency and anatomic locations of MRI abnormalities specific to limbic, circadian and motor systems in a cohort of meningoencephalitis patients, as well as to investigate the prognostic value of these MRI findings.

**Methods:**

A secondary, selective analysis of a retrospective database including all meningitis, meningoencephalitis and encephalitis cases treated between 2016 and 2018 in the University hospital of Bern, Switzerland was performed. Patients with meningitis of any cause, bacterial or autoimmune causes of encephalitis were excluded.

**Results:**

MRI scans and clinical data from 129 meningoencephalitis cases found that the most frequent causes were tick-borne encephalitis (TBE, 42%), unknown pathogens (40%), VZV (7%), and HSV1 (5%). At discharge, median modified Rankin Score (mRS) was 3 (interquartile range, IQR, 1), 88% of patients had persisting signs and symptoms. After a median of 17 months, median Glasgow Outcome Score (GOS) was 5 (IQR 1), 39% of patients still had residual signs or symptoms. All patients with HSV, 27% with TBE and 31% of those with meningoencephalitis of unknown etiology had fluid-attenuated inversion recovery (FLAIR) and to a lesser extent diffusion-weighted imaging (DWI) lesions in their initial MRI, with highly overlapping anatomical distribution. In one fifth of TBE patients the limbic system was affected. Worse outcome was associated with presence of DWI and/or FLAIR lesions and lower normalized apparent diffusion coefficient (ADC) signal intensities.

**Conclusion:**

Presence of FLAIR lesions, restricted diffusion as well as the extent of ADC hypointensity in initial MRI are parameters which might be of prognostic value regarding the longterm clinical outcome for patients with meningoencephalitis of viral and of unknown origin. Although not described before, affection of limbic structures by TBE is possible as shown by our results: A substantial proportion of our TBE patients had FLAIR signal abnormalities in these regions.

## 1 Introduction

Infectious encephalitis is a serious disease, and mortality as well age-standardized disability-adjusted life years are increasing in regions with a high socio-demographic index (1), giving rise to a considerable socioeconomic burden (2–4). In the long term, up to 30–50% of patients experience persisting signs and symptoms (3–6). In a non-pandemic setting, the estimated annual incidence of all types of encephalitis worldwide is between 1 and 13 cases per 100,000 patient-years (7, 8). Among other factors, causative agents depend on geographic location and season (7, 9, 10), with HSV being the pathogen most often identified in Western countries (2, 7, 8). In Switzerland, tick-borne encephalitis virus (TBEV) is the most frequent pathogen causing meningoencephalitis (4, 9).

The usefulness of brain MRI in the diagnostic workup of patients with infectious encephalitis has been clearly demonstrated (11). It is particularly helpful in establishing an early diagnosis in patients with HSV encephalitis (12), based on predominant signal abnormalities in the mesial temporal lobes, inferior frontal lobes and insula in almost all affected patients (13). In contrast, abnormal MRI findings have been described in only 9–33% of patients with TBE (14–16) whereas they occur in 58% of the most severely affected patients with a meningoencephaloradiculitic course of disease (16). The thalamus, basal ganglia, cerebellum and anterior horns of the spinal cord are most frequently affected (17). So far, MRI findings in meningoencephalitis have been investigated primarily for their diagnostic value, and have not been seen as a prognostic tool—except in HSV encephalitis (13, 18). Therefore, the aim of our study was to describe MRI findings specific to limbic, circadian and motor systems in our cohort of patients with meningoencephalitis and to analyze the prognostic relevance of these MRI findings for short- and long-term outcome.

## 2 Methods

### 2.1 Study design

We performed a secondary analysis of MRI scans from a retrospective database including all patients with meningitis, meningoencephalitis and encephalitis treated in our university hospital (University hospital Bern, Switzerland) from January 2016 to October 2018. The study has been approved by the local Ethics Committee (Kantonale Ethikkommission Bern, ID 2018–01523).

### 2.2 Patients

The study population has been described in detail elsewhere (4). According to published case definitions and international guidelines [e.g., (19, 20)] meningoencephalitis was claimed for patients with signs of meningitis as well as altered consciousness and/or focal neurological symptoms and/or abnormal findings in EEG. Encephalitis was defined if patients showed altered consciousness for > 24 h with no other cause and evidence of CNS Inflammation (demonstrated by at least two of the following criteria: fever, seizures or focal neurological findings attributable to the brain parenchyma, cerebrospinal fluis (CSF) pleocytosis, CSF leucocyte count > 4 × 10^6^ cells per L, EEG findings suggestive of encephalitis and/or neuroimaging findings suggestive of encephalitis). For simplicity and because a clear distinction is often difficult, the term meningoencephalitis is used in this manuscript to refer to patients with encephalitis as well as meningoencephalitis. TBE was diagnosed if patients met the following criteria: symptoms of CNS inflammation (meningoencephalitis or encephalitis criteria), history of possible exposure or tick bites and detection of TBE-specific IgM and IgG antibodies in serum using the SERION ELISA classic FSME virus/TBE virus IgG and IgM kit (Virion\Serion, Würzburg, Germany). In cases where only IgM antibodies are detected, a follow-up sample is necessary to establish the diagnosis by showing Ig G seroconversion (20). Detection of TBE viral nucleic acid in blood by PCR or isolation of TBE virus was not done.

With a FilmArray^®^ Meningitis-Encephalitis Panel (BioFire, bioMerieux, Salt Lake City, United States) done on 200 μL of CSF an automated sample homogenization, nucleic acid extraction, reverse transcription, and nucleic acid amplification was done to look for 14 common agents of community-acquired meningoencephalitis (*Escherichia coli* *K1*, *Haemophilus influenzae*, *Listeria monocytogenes*, *Neisseria meningitidis*, *Streptococcus pneumoniae*, *Streptococcus agalactiae*, *Cytomegalovirus, Enterovirus, HSV-1/2, human Herpesvirus type 6, human Parechovirus, Varicella zoster virus (VZV)* and *Cryptococcus neoformans/gattii*). Unless no bacterial cause was found by culture or serological/CSF testing (*B. burgdorferi*: by serological testing using recomWell ELISA, Mikrogen. *T. pallidum*: by reactive VRDL in CSF and/or a positive CSF intrathecal T pallidum antibody index) and an autoimmune cause excluded, unknown etiology was claimed (4).

**Figure 1:**
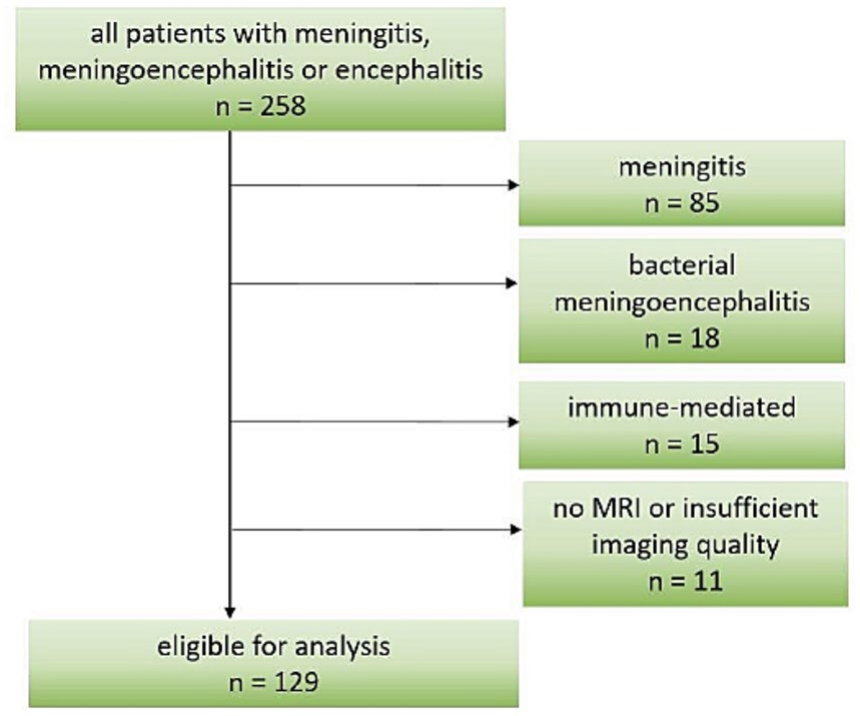
Flow chart of patient inclusion.

Given the large amount of patients with meningoencephalitis of unknown origin, they were included in our study. Important exclusion criteria were age < 16 years and primary central nervous brain MRI not performed or of poor quality. Furthermore, patients from the original study with meningitis of any cause, bacterial or autoimmune causes were excluded from the current analysis, as shown in Figure 1. Based on the available medical records, we retrospectively calculated a pre-hospital Charlson Comorbidity Index (CCI) to assess each patient’s general health status.

### 2.3 Outcome

Two neurologists assessed functional outcomes by reviewing the medical charts (U.A., A.D.) and the structured telephone interview data and questionnaires from the original study (L.S.A., A.D.) (4). The outcome was assessed at the time of hospital discharge with the mRS, and the long-term outcome by Glasgow Outcome Score (GOS, 1: death, 2: vegetative state, 3: severe deficits, 4: moderate deficits, lives autonomously 5: slight to no deficits, resumption of social and economic activities). Moreover, we aimed to classify functional outcome taking into account the persisting symptoms, the resulting limitations of everyday life and their subjectively perceived relevance. The score is described in detail in Supplementary Table S1. In brief, patients were asked about ability to return to work and to perform activities of daily life as well as the presence of remaining signs and symptoms, self-reported overall fitness and feelings of fatigue or daytime sleepiness, resulting in a score from 0 to 5. Zero indicated complete recovery, ability to return to work, no fatigue or sleep disturbances, full fitness and independence in all daily activities. Scores 1 to 4 indicated either presence of persisting signs and symptoms (1 point) and/or fatigue or disturbed nighttime sleep, rapid exhaustion after mental or physical exertion or not regained full fitness (1 point), and/or impaired functioning in one out of six daily activities (1 point) and/or unable to return to work (1 point). A score of 5 indicated death. Further outcome parameters related to persisting neurological signs and symptoms- Beck Depression Inventory II (BDI II), Fatigue Severity Scale (FSS), Epworth Sleepiness Scale (ESS) and Insomnia Severity Index (ISI) scores- collected at the follow-up interview were also considered.

### 2.4 MRI analysis

All available brain MRI acquired during the acute diagnostic workup were analyzed independently by two neurologists (L.S.A. and A.D.) and an experienced neuroradiologist (A.H.) blinded to the clinical diagnosis and patients’ outcome. MRI was acquired with 3 Tesla or 1.5 Tesla MRI units using T1-weighted, T2-weighted, susceptibility-weighted imaging (SWI) sequences, FLAIR, ADC and DWI sequences. Any disagreements about MRI findings were discussed and consensus on the findings was sought.

Signal changes in DWI and FLAIR sequences in anatomic regions related to functional systems were analyzed and a binary score was assigned for each brain region (a score of 0 if brain parenchyma was normal and 1 if brain parenchyma showed signal abnormalities). Functional systems were defined as motor areas (including gyrus precentralis, corona radiata, capsula interna, pedunculi cerebri, pons, medulla oblongata, thalamus, putamen, caudatum, pallidum, nucleus ruber, cerebellum), limbic areas (hippocampus, parahippocampal gyrus, fornix, septum pellucidum, amygdala, cingulate gyrus, anterior thalamic nuclei) and areas related to sleep–wake regulation (pons, hypothalamus, thalamus, pineal gland).

A region of interest (ROI) was drawn in the area with the most distinctive signal intensity (SI) and a signal ratio was calculated (SI in the ROI over SI of normal-appearing white matter). In patients without any discernible lesions, we recorded control ROI ratios calculating the ratio of SI in amygdala over normal-appearing white matter. Leptomeningeal enhancement was assessed by gadolinium-enhanced T1 or FLAIR according to availability. Presence and configuration (punctuate, confluent) of intra- or extra-axial hemorrhage was evaluated using SWI sequences. Presumed pre-existing lesions (e.g., those documented on previous MRI) or unrelated lesions (e.g., post stroke lesions) were documented but not included in the final analysis.

### 2.5 Statistical analysis

Statistical analysis was performed by using Stata/MP 17.0. Descriptive statistics were obtained using frequencies and percentages for categorical variables and median with interquartile range for continuous variables. Outcome analyses were performed using logistic regression for binary outcomes. The analysis includes the crude and adjusted odds ratios, where the inverse probability weighting approach, with stabilized weights, was used to perform the adjusted/weighted OR. Adjustment was made for age, sex, immunosuppression, CCI and pathogens. However, by adjusting for pathogens, results showed instability and due to convergence problems, some patients were dropped from the model. Therefore, additional analyses were done after summarizing pathogens in three groups (unknown *n* = 52, TBE *n* = 54, remaining cases *n* = 23) as well as without adjusting for causative agents. Adjustment parameters were chosen on the basis of existing literature on predictive parameters for viral meningoencephalitis (2, 21). Only complete cases were analyzed, without any multiple imputation. The estimated parameters and the 95% confidence interval are displayed in the results. Comparison of causative agents was made using the Kruskal-Wallis test for continuous variables and Fisher’s exact test for categorical variables.

## 3 Results

### 3.1 Study population

From a total of 258 patients included in the original study (4), 129 were eligible for analysis as shown in Figure 1. Demographic and clinical data are shown in Table 1. The most frequent causative pathogens were TBEV in 54 (42%), VZV in 9 (7%), and HSV1 in 7 (5%) patients. In 52 (40%) of the patients, the cause remained undetected. The median time of admission to hospital for all patients was 7 (IQR 11) days after onset of signs and symptoms: median 4 days (IQR 1) for HSV1 patients, 10 days (IQR 10) for TBE patients and 4.5 days (IQR 8.5) for patients with unknown disease etiology. The most frequent signs and symptoms were headache, fever and impaired consciousness with a documented decrease in Glasgow coma scale to below 15 in almost half the patients (Table 1). Median CCI was two points, representing a one-year mortality rate up to 26% (22).

**Table 1:** Demographic and clinical characteristics of the acute phase and follow up of all included patients.

Demographics and acute phase	*n* = 129
Gender female/male	61/68
Median age in years (IQR)	56 (32)
Median Charlson Comorbidity Index (IQR)	2 (3)
Diagnosis encephalitis/meningoencephalitis	28/101
Cause known	77 (60)
Tick-borne encephalitis virus	54 (42)
Varicella zoster virus	9 (7)
Herpes simplex virus type 1	7 (5)
Herpes simplex virus type 2	1 (1)
Enterovirus	3 (2)
Epstein–Barr Virus	2 (2)
Influenza	1 (1)
Cause unknown	52 (40)
Immunosuppression	10 (8)
Onset of signs and symptoms in median days (IQR)	7 (11)
Headache	88 (68)
Fever >38°C	81 (63)
Nuchal rigidity	29 (22)
Documented Glasgow Coma Scale <15	47 (41)
Mechanical ventilation	23 (18)
Epileptic seizure	40 (31)
Aphasia	46 (36)
Sensomotor deficits	46 (36)
Cranial nerve dysfunction	25 (19)
Median days in hospital (IQR)	10 (7)
Discharge to	
Home	41 (32)
Rehabilitation facility	59 (46)
Other Hospital for further acute care treatment	25 (19)
Death	4 (3)
Median modified Rankin Scale at discharge (IQR)	3 (1)
Any persisting signs and symptoms at discharge	114 (88)
Follow up	
Participated	79 (61)
Died/death related to meningoencephalitis	10 (8)
Declined participation	18 (14)
Lost to follow up	22 (17)
Follow up	
Follow-up interview after median months (IQR)	17 (15)
Glasgow outcome score	
5	60 (67)
4	19 (21)
1	10 (11)
Functional outcome evaluation	
Complete recovery, no fatigue, full fitness, and independence in all daily activities, return to work, score 0	27 (30)
Impairment in 1 or 2 categories, score 1–2	33 (37)
Impairment in 3 or 4 categories, score 3–4	19 (21)
Death, score 5	10 (11)
Patients with persisting neurological signs and symptoms	46 (58)
Number of persisting signs and symptoms (IQR)	
0	33 (42)
1–2	39 (49)
≥3	7 (9)
Able to return to work in former extent?	
No	16 (20)
Yes	57 (72)
N.a.^†^ (e.g., retired or no answer)	6 (8)

Unless otherwise indicated numbers and proportions (%) are given. Median and interquartile range (IQR) are given as indicated. Functional outcome evaluation (see Supplementary Table S1): additive score for impairment of one or more out of six everyday functions and/or persisting signs or symptoms and/or fatigue/excessive daytime sleepiness and/or impaired sleep quality and/or impaired fitness and/or exhaustion and/or death. ^†^Not available.

### 3.2 MRI findings

Median (IQR) time between clinical onset and initial MRI was 7 (10) days. MRI was performed on the day of admission or sometimes before transfer of the patient to our tertiary care hospital (median 0 days (IQR 1) after hospital admission). Magnetic field strength was 3 Tesla in 37% (*n* = 48) of our patients. The two neurologists and the neuroradiologist who performed blinded and independent evaluation of MRI scans did not report any major disagreements.

FLAIR signal abnormalities were seen in 30% of all patients (*n* = 38), with differences depending on the causative agent. Whereas all HSV 1 patients had FLAIR signal abnormalities, only 27% (*n* = 14) of TBE patients, 31% (*n* = 16) with unknown disease cause and one VZV patient did. No FLAIR lesions were found in the patients with HSV 2, Epstein Barr virus, enterovirus or influenza encephalitis as shown in Table 2. DWI restrictions were less frequent: overall they occurred in 15% (*n* = 19) of patients (86% of patients with HSV 1, 15% with unknown cause, 6% with TBE).

**Table 2:** MRI characteristics of all patients with encephalitis or meningoencephalitis.

	All *n* = 129	TBE *n* = 54	VZV *n* = 9	HSV1 *n* = 7	HSV2 *n* = 1	EBV *n* = 2	Enterovirus *n* = 3	Influenza *n* = 1	Unknown *n* = 52	*p*-value
MRI median days after onset of signs and symptoms (IQR)	7 (4, 14)	10 (5, 16)	8 (10)	5 (4, 5)	6	5 (1, 9)	3 (1, 5)	2	5.5 (2, 10)	0.013^¶^
Diffusion restriction (%)	19 (15)^†^	3 (6)	0 (0)	6 (86)	1 (100)	1 (50)	0 (0)	0 (0)	8 (15)	<0.001
FLAIR abnormal (%)	38 (30)^‡^	14 (27)	1 (11)	7 (100)	0 (0)	0 (0)	0 (0)	0 (0)	16 (31)	0.002
Hemorrhage^§^	11/107	2/44	1/6	2/5	1/1	0/2	0/3	0/1	5/45	0.06
Leptomeningeal enhancement^§^	49/109	20/45	3/8	3/5	NA^§§^	2/2	2/3	NA	19/46	0.63
Signs of vasculitis^§^	2/34	0/16	0/2	0/1	NA	0/1	NA	0/1	2/13	0.42

Otherwise indicated frequencies and proportions (%) are given. Columns: TBE, Tick-borne encephalitis; VZV, Varicella zoster virus; HSV1, HSV2, Herpes simplex virus type 1 and 2; EBV, Epstein–Barr virus. ^†^Diffusion weighted imaging (DWI) and apparent diffusion coefficient (ADC) available in sufficient quality in 128/129 patients. ^‡^Fluid attenuated inversion recovery (FLAIR) available in sufficient quality in 126/129 patients. ^§^only patients with adequate MRI sequences for evaluation included. ^§§^NA, not available. Statistical analysis by Fischer’s exact test unless otherwise indicated. ^¶^Kruskal-Wallis-Test.

**Figure 2:**
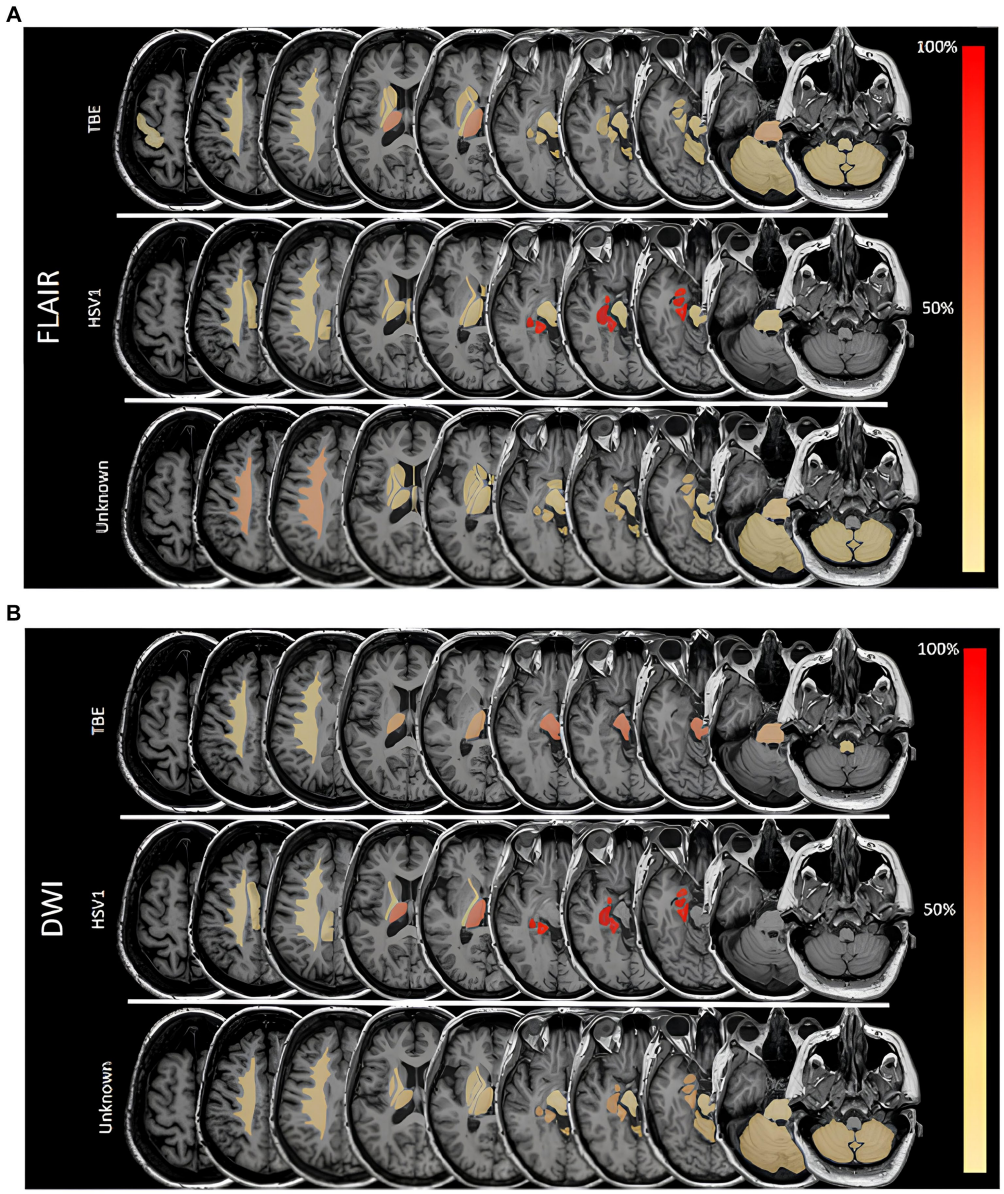
**(A)** Frequencies of lesions in fluid attenuated inversion recovery (FLAIR) sequences in different areas in patients with Herpes simplex virus type 1 (HSV1), tick-borne encephalitis (TBE) and encephalitis of unknown origin. 100% is referring to overall number of patients in a subgroup with FLAIR lesions. **(B)** Frequencies of lesions with restricted diffusion in different areas in patients with Herpes simplex virus type 1 (HSV1), tick-borne encephalitis (TBE) and encephalitis of unknown origin. 100% is referring to overall number of patients in a subgroup with restricted diffusion. DWI, diffusion weighted imaging.

As shown in Figure 2A (and Supplementary Figure S1) FLAIR lesions in TBE patients were quite dispersed, with the thalamus being the most frequently affected region (50%, *n* = 7), followed by the pontine area (29%, *n* = 4). The frequencies with which the limbic regions (amygdala and hippocampus each 21%, *n* = 3) the mesencephalon and the cerebellum (each 21%, *n* = 3) were affected were all the same. Restricted diffusion (Figure 2B) in TBE patients was found in the midbrain (67%, *n* = 2) and the pons (33%, *n* = 1).

FLAIR lesions and restricted diffusion were unilateral in all patients with HSV1 encephalitis. In the patient with encephalitis due to HSV2, restricted diffusion affected one lobe, while in patients with HSV1 encephalitis two or three cerebral lobes were involved. In patients with HSV1, FLAIR lesions and restricted diffusion were always found in the parahippocampal gyrus and hippocampus and in 86% of cases (*n* = 6) in the amygdala (Figures 2A,B). Hemorrhagic transformation was seen in 29% (*n* = 2) of patients with HSV1 encephalitis.

Patients with meningoencephalitis of unknown etiology had FLAIR lesions mostly in the corona radiata (44%, *n* = 7), but also the pontine (25%, *n* = 4) and the limbic system (hippocampus, parahippocampal gyrus, amygdala 25% each, *n* = 4). DWI restriction occurred in 15% of these patients (*n* = 8), most frequently in the limbic system (hippocampus, amygdala, parahippocampal gyrus each 38%, *n* = 3) as well as in the corona radiata, thalamus and cerebellum (each 25%, *n* = 2). Leptomeningeal enhancement was detected in 45% of all patients (*n* = 49), proportionally most frequently in those with EBV (100%, *n* = 2), EV (67%, *n* = 2) and HSV 1 (60%, *n* = 3).

Intracerebral hemorrhage occurred in 11 patients, of whom nine had punctuate intra-axial bleeding, one had confluent hippocampal bleeding associated with HSV1 and one had a subarachnoid hemorrhage. The locations of the punctate intra-axial bleeding were: Hippocampus (*n* = 1), amygdala (*n* = 1), thalamus (*n* = 2), insula (*n* = 1), putamen (*n* = 1), mesencephalon (*n* = 1), pons (*n* = 2). Two patients showed signs of vasculitis, meningoencephalitis was of unknown cause in both of them. However, evaluation of vasculitis was possible in only 34 of patients (27%) due to frequent lack of adequate MRI sequences.

FLAIR lesions considered unrelated to the acute disease were found in 57 (45%) patients and were classified as post-stroke (*n* = 5), post-surgery (*n* = 2), multiple sclerosis (*n* = 1), microvascular (*n* = 19), nonspecific deep white matter changes (*n* = 27) and unclear (*n* = 6).

### 3.3 Outcome

Patients were discharged after a median hospital stay of 10 days and 65% (*n* = 84) of them were transferred to another hospital for further acute treatment or to a rehabilitation unit. Four patients died (two with TBE, one with EBV infection and one with unknown disease etiology), 32% (*n* = 41) of patients were discharged home. At discharge, median mRS was 3 (IQR 1) and 88% of patients (*n* = 114) still had persisting signs and symptoms of the acute disease.

A median of 17 months after hospital discharge, a cross-sectional telephone interview was performed. Data were available for 79 patients; ten patients had died in the meantime (Table 1). At follow-up, median GOS was 5 (IQR 1) (Table 1), 58% (*n* = 46) of patients still complained of residual symptoms. Mean functional outcome evaluation score was 2 (IQR 3) (Table 1). A detailed description of clinical sequelae has been published elsewhere (4); a summary is provided in Table 1.

In our assessment of MRI findings and outcome we found a possible association of worse outcome (GOS) with presence of diffusion restriction and/or FLAIR hyperintense lesions in the initial MRI, as shown in Table 3A. Patients without restricted diffusion/FLAIR lesions were more likely to be able to return to work in their former capacity. Moreover, the risk of a poor functional outcome score increased, the lower the normalized apparent diffusion coefficient (ADC) SI of an affected region (i.e., hypointense ADC). Furthermore, the more anatomical regions were affected in both DWI and FLAIR sequences, the worse the outcome. Presence of FLAIR hyperintensities was associated with poorer long-term outcome (GOS, impossible to work in former extent) in the two largest etiologic subgroups (TBE and meningoencephalitis of unknown cause) as shown in Tables 3B,C.

**Table 3:** Association of abnormal MRI findings with measures of poor functional outcome, adjusted results.

(A) Overall population										
	DWI restriction OR^†^ (95%-CI), IPW^*^	*p*-value	FLAIR hyperintensity OR (95%-CI), IPW	*p*-value	Greater ADC ratio OR (95%-CI), IPW	*p*-value	Leptomeningeal enhancement OR (95%-CI), IPW	*p*-value	Hemorrhagic signs OR (95%-CI) IPW	*p*-value
Poor mRS^‡^ at discharge	1.68 (0.57–4.95)	0.347	1.53 (0.70–3.32)	0.284	0.98 (0.95–1.02)	0.336	1.37 (0.63–2.97)	0.425	0.17 (0.04–0.70)	0.014
GOS^§^ poor (1–4)	8.47 (1.71–41.80)	0.009	8.20 (2.43–27.60)	0.001	0.96 (0.91–1.01)	0.114	1.44 (0.41–5.02)	0.571	4.05 (0.57–28.77)	0.162
Impossible to return to work	11.32 (1.78–71.98)	0.010	8.38 (2.25–31.19)	0.002	0.97 (0.92–1.02)	0.195	0.93 (0.24–3.56)	0.915	4.23 (0.59–30.32)	0.151
Poor functional outcome score	6.17 (1.25–30.57)	0.026	2.55 (0.87–7.45)	0.087	0.95 (0.91–1.00)	0.040	1.06 (0.41–2.73)	0.911	2.14 (0.34–13.38)	0.416

(B) Patients with tick-borne encephalitis and meningoencephalitis of unknown cause and a poor Glasgow Outcome Scale (1–4)										
	DWI restriction OR^†^ (95%-CI), IPW	*p*-value	FLAIR hyperintensity OR^†^ (95%-CI), IPW	*p*-value	Greater ADC ratio OR (95%-CI), IPW	*p*-value	Leptomeningeal enhancement OR (95%-CI), IPW	*p*-value	Hemorrhagic signs OR (95%-CI) IPW	*p*-value
TBE^‡^	3.93 (0.21–74.26)	0.362	12.41 (1.64–93.96)	0.015	0.89 (0.78–1.02)	0.097	1.45 (0.19–10.85)	0.717	N.D.**	N.D.
ME^§^ of unknown etiology	12.17 (0.74–199.17)	0.080	38.43 (2.57–574.51)	0.008	1.02 (0.94–1.11)	0.686	0.33 (0.03–3.79)	0.374	N.D.	N.D.

(C) Patients with tick-borne encephalitis and meningoencephalitis of unknown cause, inable to work in former extent										
	DWI restriction OR^†^ (95%-CI), IPW	*p*-value	FLAIR hyperintensity OR^†^ (95%-CI), IPW	*p*-value	Greater ADC ratio OR (95%-CI), IPW	*p*-value	Leptomeningeal enhancement OR (95%-CI), IPW	*p*-value	Hemorrhagic signs OR (95%-CI) IPW	*p*-value
TBE^‡^	4.13 (0.21–79.84)	0.384	5.75 (0.73–45.17)	0.096	0.92 (0.80–1.04)	0.188	0.98 (0.11–8.78)	0.989	N.D.	N.D.
ME^§^ of unknown etiology	8.61 (0.40–186.93)	0.170	31.43 (1.98–499.92)	0.015	1.05 (0.94–1.18)	0.360	N.D.	N.D.	N.D.	N.D.

(A) MRI sequences: DWI, Diffusion weighted imaging; FLAIR, fluid attenuated inversion recovery; ADC, apparent diffusion coefficient. ^†^Adjusted odds ratios (OR) for age, sex, immunosuppression and Charlson Comorbidity Index. *Inverse probability weighting. ^‡^Modified Rankin Scale. ^§^Glasgow Outcome Scale.

(B) and (C) MRI sequences: DWI, Diffusion weighted imaging; FLAIR, fluid attenuated inversion recovery; ADC, apparent diffusion coefficient. ^†^Adjusted odds ratio (OR) for age, sex, immunosuppression and Charlson Comorbidity Index. *Inverse probability weighting. ^‡^Tick-borne encephalitis. ^§^Meningoencephalitis. **Not possible to derive.

We found no indication of an association between DWI/FLAIR ROI ratios, leptomeningeal enhancement or hemorrhagic signs in initial MRI and immediate or long-term clinical outcome. No association was found between MRI variables and BDI II, FSS, ESS or ISI.

## 4 Discussion

In our study on 129 patients with meningoencephalitis, we found signal abnormalities in up to 30% of patients. All patients with HSV encephalitis, but only 27% of those with TBE and 31% of patients with meningoencephalitis of unknown cause had FLAIR signal abnormalities. Temporal lobe was a predilection site of MR lesions in HSV encephalitis patients, in line with existing literature (13, 23, 24). However, TBE patients had lesions in more dispersed brain regions, most importantly the thalamus and pontine but also in limbic regions as well as the mesencephalon and cerebellum (Figures 2A,B). In patients with meningoencephalitis of unknown etiology lesions occurred predominantly in the corona radiata, pons and the limbic system.

The signal abnormalities in limbic areas seen in TBE patients have not previously been described (14, 17, 25, 26) and highlight the low specificity of MRI findings regarding the etiology of meningoencephalitis in the acute phase of this disease. Our findings of effects on the thalamus and brainstem in patients with TBE are in line with the literature (15–17, 27, 28). Our findings that temporal lobes are always affected by FLAIR and DWI lesions in in patients with HSV 1 encephalitis, are perfectly in line with other studies investigating larger cohorts (13, 23, 24, 29). Likewise, our findings on TBE are consonant with published studies reporting frequencies of MRI lesions ranging from 9% up to >58% (14–16, 26, 30). One reason for our comparably high rate of parenchymal MRI changes could be the timing of MRI acquisition: the median time to acquisition of MRI was 10 days after symptom onset, corresponding to the time of maximal inflammatory changes with optimal detectability of IgM/G in cerebrospinal fluid and of viral antigens in the brain (28, 31). The timing of MRI acquisition in relation to symptom onset is essential information and its absence limits the validity of results reported in some studies (15, 16, 30). Furthermore, magnetic field strength was 3 Tesla in more than one third of our patients. It is possible that earlier studies were performed mainly with 1.5 Tesla MRI, increasing the risk of overlooked lesions. However, most studies did not report magnetic field strengths. In a recent study on 52 TBE patients including MRI data, the vaccination breakthrough cases were more likely to have more extensive MRI lesions (26). Unfortunately, we had no information on the vaccination status of our patients.

Regarding prognosis, presence of restricted diffusion and FLAIR lesions in the initial MRI was not associated with mRS at discharge in the overall population. However, there was an association with long-term outcome: The GOS was associated with the presence of DWI and FLAIR lesions and restricted diffusion. Also, in the analysis of the two largest etiologic subgroups (TBE and disease of unknown disease cause), absence of FLAIR lesions correlated with a better GOS and with a higher probability to take up work in former extent (unknown disease cause).

A poorer functional outcome score was associated with a lower normalized ADC SI of an affected region. On one hand, this reflects that patients with a cytotoxic lesion pattern have a worse long-term functional outcome. We found one study with similar results, where more severe acute disease course in HSV1 patients was found to be correlated with cytotoxic lesion pattern (ADC 0.41 × 10^−3^ mm^2^/s) rather than with a vasogenic (ADC 1.43 × 10^−3^ mm^2^/s) one (32). Our results further suggest that the lower the ADC value in a cytotoxic lesion, the worse the functional long-term outcome.

Due to the small amount of patients, we did not perform an outcome analysis for subgroups other than meningoencephalitis due to TBEV or unknown origin. Literature on MRI findings and prognosis in patients with TBE is sparse and contradictory: No association was found between T2 lesions in initial MRI (9 days after symptom onset) and mortality until 3 months after discharge (14). There was also no correlation between initial MRI findings (acquisition time and sequences not specified) and clinical outcome measured by mRS 1 year after hospital discharge (16) or the support group questionnaire ESGQ 5.5 years after hospital discharge (30). However, one large study described an association between MRI abnormalities (not further specified) and long-term clinical outcome after 1 year (15).

The proportion of patients with unidentified causes of meningoencephalitis often remains high despite thorough diagnostic workup. In line with published cohort studies (2, 7, 33, 34) disease etiology remained undetermined in 40% of our cases. In contrast to our results for this subgroup, however, other authors who conducted small (*n* = 11) and middle-sized (*n* = 59) studies did not describe any association of MRI lesions with overall clinical outcome (35, 36). This difference might be attributable to the slightly higher proportion of patients with FLAIR abnormalities in our subgroup [31% vs. 27% in (35)] and the shorter follow-up time in our subgroup [17 months vs. 32 months (36)].

For patients with HSV encephalitis other studies have found an association of more extensive brain lesions (i.e., bilateral DWI lesions and FLAIR lesions affecting more than three brain lobes) with worse outcome (13). In adult patients with encephalitis caused by VZV, EBV, EV, or influenza, existing literature does not permit a conclusion on the prognostic value of MRI findings as most studies report mainly on findings in children [e.g., (37)].

Leptomeningeal enhancement was found in 45% of all patients with meningoencephalitis whatever the cause. It was the most frequent finding in patients with meningoencephalitis of unknown etiology and those with TBE (41 and 44% respectively). The rate of leptomeningeal enhancement in TBE patients was in line with histopathological studies, where inflammatory changes were predominantly seen in meninges (28) and with another study where one third of patients had leptomeningeal enhancement (26). However, frequency of leptomeningeal enhancement is often not described in literature or else no distinction is made between parenchymal and leptomeningeal enhancement. In our study, presence of leptomeningeal enhancement was not associated with short- or long-term clinical outcome.

Given the often highly overlapping distribution of lesions, we assume that this parameter is of limited usefulness in differentiating causative pathogens in patients with meningoencephalitis.

Comparable to previous works, a limitation of our study was the large number of patients with meningoencephalitis of unknown etiology. We defused the situation by characterizing this subgroup as precisely as possible and by performing subgroup analyses. Further limitations were the retrospective design and the partly small group sizes. Our study was monocentric and undertaken in a tertiary hospital; facts that should be considered when applying our results to other patients. Furthermore, it was sometimes challenging to distinguish FLAIR lesions due to meningoencephalitis from pre-existing ones. Lesions without involvement of the predefined anatomical locations were not considered and outcome data was available for a relatively small number of patients, potentially leading to selection bias: Overestimation of good outcomes is possible because patients lost to follow-up were probably those with a particularly bad outcome who were not able to take part in a telephone interview. Clinical outcomes were partially self-reported and this might have further biased results. In this study we focused on the prognostic value of MRI variables. As it has been shown elsewhere [e.g., (2, 21)] demographic and clinical factors are of prognostic relevance too.

## 5 Conclusion

We found a possible association between presence of restricted diffusion and particularly for the presence of FLAIR lesions in initial MRI and a worse overall clinical long-term outcome measured by GOS. Presence of FLAIR lesions in initial MRI might be of prognostic value also for patients with meningoencephalitis of unknown cause. More hypointense ADC values might increase the risk of a poorer functional outcome. A substantial proportion of TBE patients had FLAIR signal abnormalities in limbic regions.

## Data availability statement

The raw data supporting the conclusions of this article will be made available by the authors, without undue reservation.

## Ethics statement

The studies involving humans were approved by the Kantonale Ethikkommission Bern, ID 2018–01523. The studies were conducted in accordance with the local legislation and institutional requirements. Written informed consent for participation in this study was provided by the participants’ legal guardians/next of kin.

## Author contributions

LA: Conceptualization, Data curation, Investigation, Methodology, Project administration, Visualization, Writing – original draft, Writing – review & editing. MB: Conceptualization, Data curation, Formal analysis, Methodology, Writing – review & editing. AU: Data curation, Writing – review & editing. AF: Formal analysis, Writing – review & editing. SL: Writing – review & editing. CB: Supervision, Writing – review & editing. AH: Conceptualization, Data curation, Investigation, Methodology, Supervision, Visualization, Writing – review & editing. AD: Conceptualization, Data curation, Formal analysis, Investigation, Methodology, Supervision, Visualization, Writing – review & editing.
