# Supplementary material for: Magnetic resonance imaging in acute meningoencephalitis of viral and unknown origin: frequent findings and prognostic potential
Source: Front Neurol. 2024 Jan 17;15:1359437. doi: 10.3389/fneur.2024.1359437 (PMC10829495; doi:10.3389/fneur.2024.1359437)
Supplement: Supplementary file 1 [file Table_1.DOCX]

**Supplementary table 1:** Functional outcome score (FOS)

| 1. DAILY ACTIVITY   1 point if one or more identified   - Inability to use a phone? - Inability to cook a meal? - Inability to use public transport? - Inability to wash the laundry? - Inability to do the grocery - Inability to handle financial matters? | 1. CIRCADIAN RHYTHM/FATIGUE   1 point if one or more identified   - New onset daytime sleepiness&/or fatigue - New onset disturbed night time sleep - New onset quick exhaustion after physical or mental activity - Feeling not completely recovererd |
| --- | --- |
| 1. NEUROLOGICAL SYMPTOMS   1 point if suffering from persisting neurological symptoms since acute disease. | 1. WORK LIFE   1 point if unable to return to work in former extent. |
| 1. DEATH   5 points if patient died from acute (meningo)-encephalitis. | *To get FOS (0-5)*  Add up points from categories A-D  or mark 5 if patient died. |

Good outcome = total sum score 0-1. Poor outcome= total sum score 2-5.
