# Supplementary material for: Magnetic resonance imaging in acute meningoencephalitis of viral and unknown origin: frequent findings and prognostic potential
Source: Front Neurol. 2024 Jan 17;15:1359437. doi: 10.3389/fneur.2024.1359437 (PMC10829495; doi:10.3389/fneur.2024.1359437)
Supplement: Supplementary file 2 [file Table_2.DOCX]

**Supplementary table 2:** Association of abnormal MRI findings with measures of poor functional outcome, additionally adjusted for causative agents

Overall population

|  | DWI restriction adjusted OR (95%-CI) | p-value | FLAIR hyperintensity adjusted OR (95%-CI) | p-value | greater ADC ratio OR (95%-CI) | p-value | Leptomeningeal enhancement OR (95%-CI) | p-value | Hemorrhagic signs OR (95%-CI) | p-value |
| --- | --- | --- | --- | --- | --- | --- | --- | --- | --- | --- |
| Poor mRS ^‡^ at discharge | *0.98 (0.25-3.81)  **1.11 (0.32-3.87) | *0.979  **0.869 | *1.40 (0.59-3.31)  **1.46 (0.67-3.18) | *0.445  **0.338 | *0.99 (0.96-1.03)  **0.98 (0.95-1.02) | *0.649  **0.337 | *1.23 (0.56-2.70)  **1.39 (0.64-3.03) | *0.605  **0.404 | *0.40 (0.08-1.98)  **0.38 (0.08-1.77) | *0.259  **0.217 |
| GOS^§^ poor (1-4) | *7.60 (1.11-52.24) **6.96 (1.10-44.16) | *0.039  **0.039 | *14.91 (3.55-62.59)  **8.24 (2.44-27.77) | *< 0.001  **0.001 | *0.95 (0.90-1.01)  **0.95 (0.90-1.00) | *0.075  **0.056 | *1.16 (0.32-4.23)  **1.41 (0.4-4.98) | *0.827  **0.598 | *0.43 (0.04-4.75)  **1.50 (0.21-10.60) | *0.494  **0.684 |
| Impossible to return to work | *7.49 (0.96-58.67)  **8.19 (1.12-59.95) | *0.055  **0.038 | *11.42 (2.47-52.66)  **8.29 (2.23-30.87) | *0.002  **0.002 | *0.96 (0.91-1.02)  **0.95 (0.90-1.01) | *0.200  **0.095 | *0.65 (0.16-2.69)  **0.87 (0.22-3.37) | *0.549  **0.842 | *0.45 (0.04-5.01)  **1.55 (0.22-11.04) | *0.519  **0.662 |
| Poor functional outcome score | *6.08 (0.77-48.23)  **4.25 (0.59-30.72) | *0.088  **0.152 | *4.81 (1.38-16.81)  **2.55 (0.87-7.45) | *0.014  **0.087 | *0.94 (0.90-0.99)  **0.95 (0.91-1.00) | *0.025  **0.041 | *0.96 (0.37-2.52)  **1.08 (0.41-2.79) | *0.938  **0.881 | *1.25 (0.17-9.03)  **1.52 (0.24-9.78) | *0.822  **0.660 |

Table legend: MRI sequences: Diffusion weighted imaging (DWI), fluid attenuated inversion recovery (FLAIR), apparent diffusion coefficient (ADC). Adjusted odds ratios (OR) for age, sex, immunosuppression, Charlson Comorbidity Index and causative agents considered * individually and summarized in **three groups (groups: unknown, tick-borne encephalitis, remaining pathogens). ^‡^Modified Rankin Scale. ^§^ Glasgow Outcome Scale.
