# Supplementary material for: Magnetic resonance imaging in acute meningoencephalitis of viral and unknown origin: frequent findings and prognostic potential
Source: Front Neurol. 2024 Jan 17;15:1359437. doi: 10.3389/fneur.2024.1359437 (PMC10829495; doi:10.3389/fneur.2024.1359437)
Supplement: Supplementary file 3 [file Table_3.DOCX]

**Supplementary table 3**: Association of abnormal MRI findings with measures of poor functional outcome, unadjusted values

1. Overall population

|  | DWI restriction  OR ^†^ (95%-CI) | p-  value | FLAIR hyperintensity  OR (95%-CI) | p-  value | greater ADC ratio  OR (95%-CI) | p-  value | leptomeningeal enhancement  OR (95%-CI) | p-  value | hemorrhagic signs  OR (95%-CI) | p-  value |
| --- | --- | --- | --- | --- | --- | --- | --- | --- | --- | --- |
| Poor mRS ^‡^ at discharge | \| 2.29 (0.81-6.46) \| 0.118 \| \| --- \| --- \| \|  \| 0.005 \| \|  \| 0.005 \| \|  \| 0.050 \| | \| 0.118 \| \| --- \| \|  \| | \| 1.51 (0.70-3.25) \| \| --- \| \|  \| \|  \| \|  \| | \| 0.296 \| \| --- \| | \| 0.98 (0.95-1.01) \| \| --- \| \|  \| \|  \| | \| 0.219 \| \| --- \| \|  \| | \| 1.21 (0.57-2.57) \| \| --- \| \|  \| \|  \| \|  \| | 0.623 | 0.35 (0.09-1.38) | 0.132 |
| GOS^§^ poor (1-4) | 8.62 (1.90-39.06) | 0.005 | 8.10 (2.44-26.82) | 0.001 | 0.96 (0.91-1.01) | 0.110 | 1.57 (0.47-5.31) | 0.466 | 2.36 (0.36-15.65) | 0.374 |
| Impossible to return to work | 12.27 (2.10-71.57) | 0.005 | 8.17 (2.24-29.84) | 0.001 | 0.97 (0.92-1.02) | 0.207 | 1.10 (0.30-4.07) | 0.888 | 2.44 (0.37-16.34) | 0.356 |
| Poor functional outcome score | 4.81 (1.00-23.21) | 0.050 | 2.43 (0.84-7.00) | 0.099 | 0.95 (0.91-1.00) | 0.034 | 1.06 (0.42-2.68) | 0.898 | 1.35 (0.23-7.90) | 0.739 |

Table legend: MRI sequences: Diffusion weighted imaging (DWI), fluid attenuated inversion recovery (FLAIR), apparent diffusion coefficient (ADC). ^†^ Unadjusted odds ratios (OR) ^‡^Modified Rankin Scale. ^§^ Glasgow Outcome Scale.

1. Patients with tick-borne encephalitis & meningoencephalitis of unknown cause

|  | DWI restriction  OR ^†^ (95%-CI) | p-  value | FLAIR hyperintensity  OR (95%-CI) | p-  value | greater ADC ratio  OR (95%-CI) | p-  value |
| --- | --- | --- | --- | --- | --- | --- |
| Poor mRS ^‡^ at discharge | TBE: 0.44 (0.04-5.22)  UC: 3.60 (0.65-19.84)   \|  \| 0.141 \| \| --- \| --- \| | TBE: 0.519  UC: 0.141 | TBE: 0.61 (0.18-2.09)  UC: 1.53 (0.46-5.02) | TBE: 0.429  UC: 0.486 | TBE: 0.98 (0.93-1.04)  UC: 0.99 (0.94-1.04) | TBE: 0.554  UC: 0.760 |
| GOS^§^ poor (1-4) | TBE: 3.86 (0.21-69.67)  UC: 15.33 (1.05-224.78) | TBE: 0.361  UC: 0.046 | TBE: 8.00 (1.28-50.04)  UC: 42.00 (3.03-581.43) | TBE: 0.026  UC: 0.005 | TBE: 0.88 (0.78-1.00)  UC: 0.99 (0.92-1.06) | TBE: 0.051  UC: 0.795 |
| Impossible to return to work | TBE: 4.17 (0.23-76.60)  UC: 7.67 (0.37-157.36) | TBE: 0.337  UC: 0.186 | TBE: 5.50 (0.80-37.61)  UC:31.50 (2.14-463.14) | TBE: 0.082  UC: 0.012 | TBE: 0.90 (0.79-1.01)  UC: 1.03 (0.92-1.14) | TBE: 0.074  UC: 0.620 |
| Poor functional outcome score | TBE: 2.00 (0.17-24.07)  UC: * N.D. | TBE: 0.585  UC: N.D. | TBE: 2.69 (0.58-12.60)  UC: 7.58 (0.81-71.05) | TBE: 0.208  UC: 0.076 | TBE: 0.95 (0.88-1.02)  UC: 0.96 (0.89-1.03) | TBE: 0.139  UC: 0.222 |

Table legend: MRI sequences: Diffusion weighted imaging (DWI), fluid attenuated inversion recovery (FLAIR), apparent diffusion coefficient (ADC). Tick-borne encephalitis (TBE), unknown cause (UC). * Not possible to derive. ^†^ Unadjusted odds ratios (OR) ^‡^Modified Rankin Scale. ^§^ Glasgow Outcome Scale.
