# Supplementary figures and images for: Magnetic resonance imaging in acute meningoencephalitis of viral and unknown origin: frequent findings and prognostic potential
Source: Front Neurol. 2024 Jan 17;15:1359437. doi: 10.3389/fneur.2024.1359437 (PMC10829495; doi:10.3389/fneur.2024.1359437)

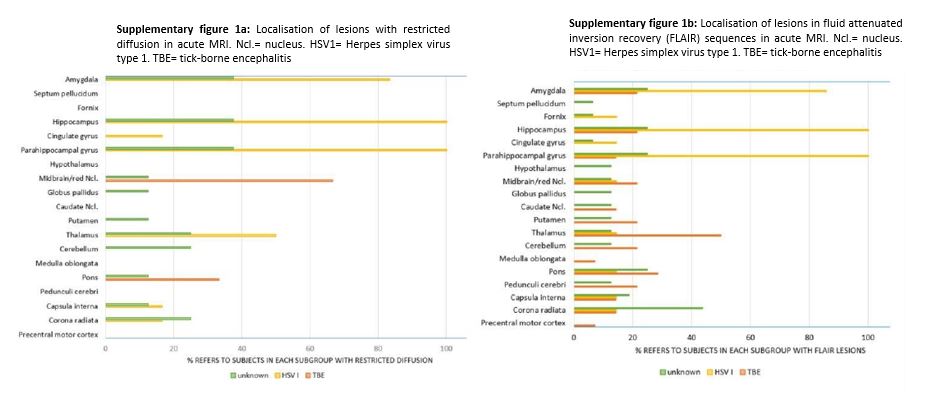

Supplement: Supplementary file 4 [file Image_1.JPEG]
